# Disentangling the drivers of diversification in an imperiled group of freshwater fishes (Cyprinodontiformes: Goodeidae)

**DOI:** 10.1186/s12862-018-1220-3

**Published:** 2018-07-18

**Authors:** Kimberly L. Foster, Kyle R. Piller

**Affiliations:** 1 Department of Biological Sciences, Southeastern Louisiana University, Hammond, LA 70402 USA; 2 Present Address: Department of Biological Sciences, Western Michigan University, Kalamazoo, MI 49008-5410 USA

**Keywords:** Ecological opportunity, Diversification, Fishes, Mexico, Body shape, Phylogenetics

## Abstract

**Background:**

One of the most perplexing questions in evolutionary biology is why some lineages diversify into many species, and others do not. In many cases, ecological opportunity has played an important role, leading to diversification along trophic or habitat-based axes. The Goodeidae (Teleostomi: Cyprinodontiformes) are a family of freshwater fishes with two subfamilies: Goodeinae (42 species, viviparous, heterogeneous habitats, Mesa Central of Mexico) and Empetrichthyinae (4 species, oviparous, homogeneous habitats, Great Basin of the United States). These discrepant sets of characteristics and their sister-group relationship make the goodeids amenable to a comparative study of diversification. We gathered lateral body images from more than 1600 specimens of all extant species in the family. Geometric morphometric, and phylogenetic comparative analyses were used to address whether higher species diversity correlates with higher rates of morphological shape evolution and whether there are differences in functional/habitat modules between the two subfamilies.

**Results:**

This study recovered a higher rate of overall body shape evolution in the Goodeinae that is nearly double in magnitude compared to the Empetrichthyinae. A modularity test indicated that the Goodeinae displayed elevated rates of morphological evolution in comparison to the Empetrichthyinae when only trunk (locomotor) regions were compared between subfamilies. No significant differences in evolutionary shape rates were recovered when the trophic (head) regions were compared between subfamilies.

**Discussion:**

These results support the hypothesis that Mexican goodeids radiated via an ecological opportunity scenario into a wide-array of novel habitats in the island-like Mesa Central as evidenced by their high rate of shape evolution, relative to the Empetrichthyinae. This study quantitatively unraveled the drivers of evolution and eliminated trophic specialization as a driving force within the Goodeidae.

**Conclusions:**

A combination of phylogenetic and morphometric data, and phylogenetic comparative analyses were used to examine body shape rate evolution within the Goodeidae. Results support the hypothesis that species in the subfamily Goodeinae on the central Mexican plateau had a higher rate of body shape evolution relative to its sister subfamily Empetrichthyinae in the Great Basin suggesting that the Goodeinae diversified via an ecological opportunity scenario along habitat, rather than trophic axes.

**Electronic supplementary material:**

The online version of this article (10.1186/s12862-018-1220-3) contains supplementary material, which is available to authorized users.

## Background

Disproportionate species richness among clades is one of the most interesting patterns in evolutionary biology, with some clades being exceedingly diverse while others are relatively depauperate [1–3]. The drivers of speciation and diversification that result in clade imbalance have been the subject of an ongoing discussion within the literature for decades [4–9]. Although species richness might be expected to vary purely by stochastic processes, many other ideas have been put forth to explain discrepant patterns. First, clade age is believed to be an important factor, whereas older clades are expected to have higher species richness due to the greater length of time for diversification and speciation to occur [10, 11]. Second, it has been shown that differential diversification rates are a common explanation for clade disparity [3, 12, 13]. Differential speciation and extinction rates are often correlated with phenomena such as key innovations, adaptive radiations, which can increase diversification rates, and contrasting geologic and climatic histories, which can increase extinction rates [14–17]. In fact, the majority of the most heavily studied examples of adaptive radiation are related to trophic and/or habitat specializations [18–22].

It is generally expected that species-rich clades should harbor higher levels of phenotypic diversity in comparison to less speciose clades [23, 24]. Morphological disparity and speciation may be linked, and one hypothesis suggests that clades with higher rates of phenotypic evolution may be able to reach into novel ecological trait space, leading to an increase in diversification [24–26]. The idea that some organisms are morphologically more versatile than others leading to replacement of the latter is a key concept regarding ecological opportunity and adaptive zones [23, 27, 28].

**Fig. 1:**
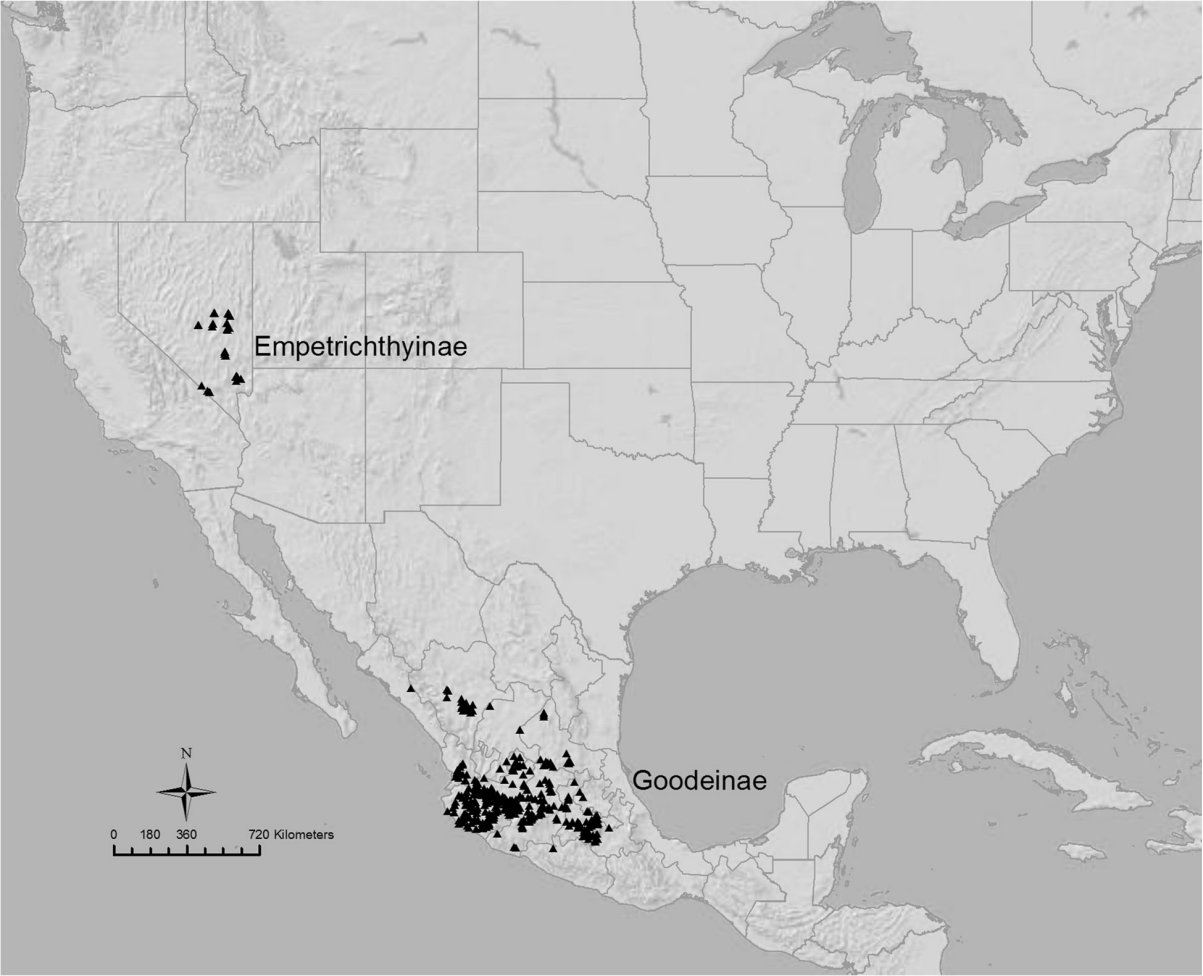
Distribution of the two subfamilies of the Goodeidae based on vouchered museum records (http://fishnet2.net/, May 2017)

The freshwater fish family Goodeidae (Order: Cyprinodontiformes) [29] is an ideal group to address the process of diversification. The family is found within the Great Basin of the southwestern United States and the central Mexican highlands (Fig. 1). This disjunct distributional pattern is unique to Goodeidae, and found in no other primary freshwater fish groups. Increasing desiccation of the Sonoran Desert during the Tertiary is hypothesized to have divided the ancestral goodeid lineage into two disjunct subfamilies [30, 31], the Goodeinae [29] and the Empetrichthyinae [32].

The subfamily Goodeinae is endemic to Mexico, with approximately 18 genera and 42 extant species, with the highest diversity occurring in the geographic area known as the Mesa Central, a relatively depauperate, isolated highland plateau [33–35]. This region has been subjected to substantial volcanic and tectonic activity since the beginning of the Miocene, leading to intricate hydrological systems [36, 37], which have likely contributed to higher isolation and speciation in this area [35]. All species in the subfamily are viviparous, and embryos of all species possess a vascular rectal structure, known as a trophotaeniae, for nutrient absorption [38–40]. Across the Goodeinae, the species possess trophic ecologies ranging from strictly carnivorous (*Alloophorus robustus* and *Allodontichthys tamazulae*) to herbivorous (*Goodea atripinnis*), however, the majority of species possess similar diets, are omnivorous, and occupy different niches within the trophic spectrum between these two dietary extremes [35, 41, 42]. Species of Goodeinae inhabit lakes, creeks, marshes, canals, and large rivers [35], with some species being habitat specialists (i.e. springs only) and others being more generalist in terms of their habitat preferences.

The sister group to the Goodeinae, the subfamily Empetrichthyinae is much less diverse than the Goodeinae. Two genera, *Crenichthys* and *Empetrichthys*, and three species (and multiple subspecies) currently occupy the Great Basin of the United States. Three additional taxa have gone extinct within the last century [42–44]. All species of Empetrichthyinae are oviparous, are opportunistic omnivores and utilize similar niches in springs and pools of the Great Basin of the United States [44, 45].

The disparities between the two subfamilies within the Goodeidae offer an excellent opportunity to test for contributions to lineage diversification. In addition to the distinct morphological and life-history differences, one important advantage of the chosen taxa is that they are sister lineages, which therefore allows for the removal of the clade-age effect from the comparison. The objectives of this paper are two-fold. First, to determine whether there are differences in rates of multivariate morphological evolution between the two clades, and whether those differences reflect the hypothesis that the Goodeinae, due to their higher level of diversity and range of morphological variation, reach into novel areas of morphological trait space. Second, to test for differences in rates of evolution between phenotypic modules for the two groups. As stated earlier, trophic specialization and occupancy of new niches are most often associated with elevated speciation rates in other groups, particularly in adaptive radiation scenarios, which has been hypothesized for the Goodeinae on the Mesa Central [31]. In this comparison, it is expected that the head regions of the Goodeinae will vary at higher evolutionary rates if trophic specialization is the selective force (*trophic diversification hypothesis*). Alternatively, if the trunk region of the Goodeinae display elevated evolutionary rates relative to the Empetrichthyinae, this would indicate that the observed patterns are more likely the result of the occupancy of novel habitats and the associated hydrological constraints during the radiation (*habitat diversification hypothesis*).

## Results

### Phylogenetic analysis

**Fig. 2:**
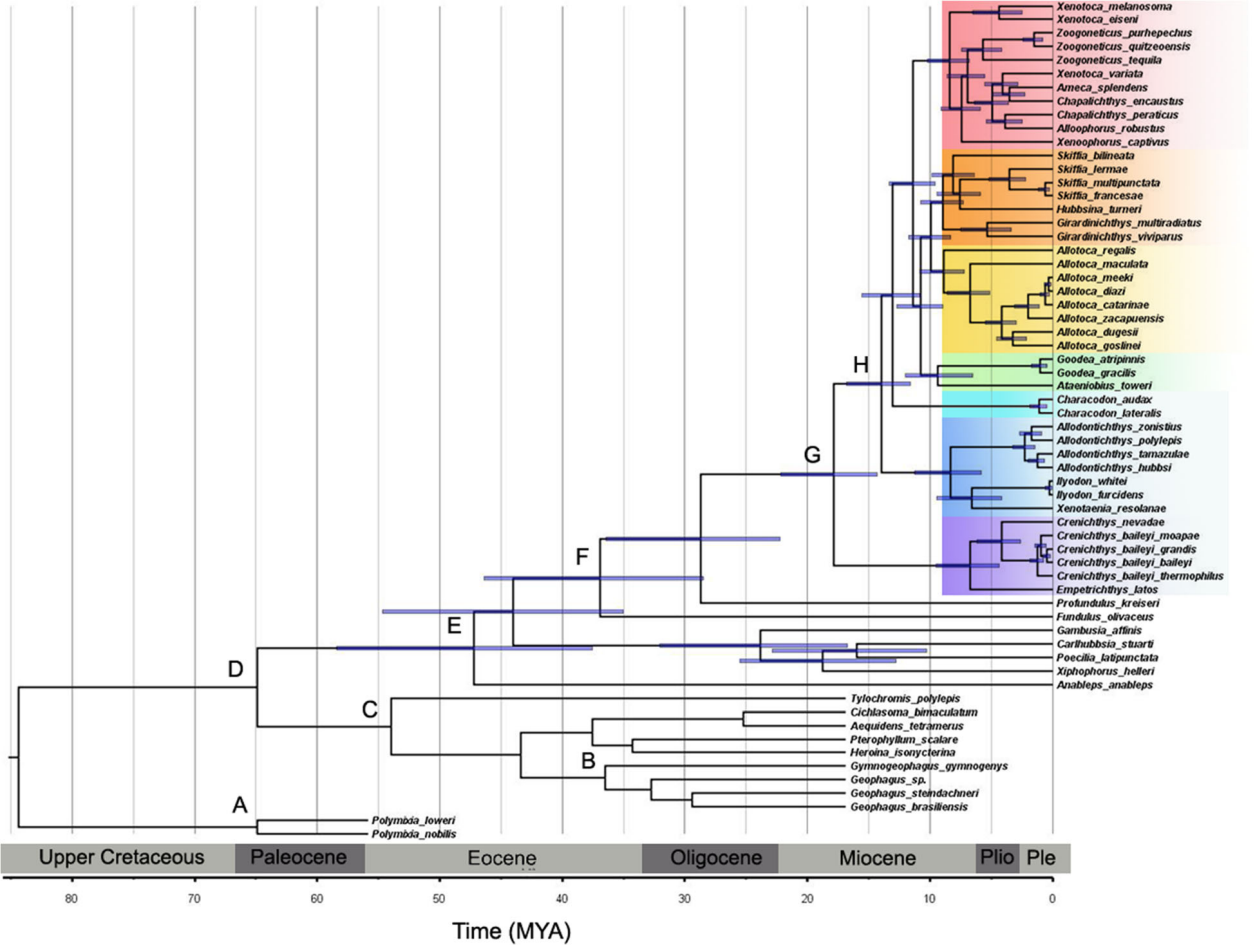
Time-calibrated phylogeny generated in BEAST using cytochrome *b* data from Doadrio and Dominguez [33], and additional taxa of Empetrichthyinae sequenced in this study. Fossil calibrations are shown at each node and referenced in Table 3

The results of the phylogenetic analysis are similar to those of [33] (Fig. 2), but this present study included additional empetrichthyine taxa not included by those authors. The phylogenetic analysis resulted in a sister group relationship between Goodeinae and Empetrichthyinae. A monophyletic clade was inferred that consisted of all the subspecies of *Crenichthys baileyi* and *Crenichthys nevadae*, and this clade was sister to *Empetrichthys latos*, the sole extant species in this genus. Profundulidae is supported as the sister family to Goodeidae. The split of Empetrichthyinae and Goodeinae is estimated at 18.02 Mya (14.3–22.17 Mya). The tribes within Goodeinae diverged around 14.05 Mya (11.62–16.79). The separation of the genera *Empetrichthys* and *Crenichthys* is estimated at 6.88 Mya (4.35–9.48 Mya) (Table 1).

**Table 1:** Major divergence time estimates with the Goodeidae from the BEAST dating analysis

Cladogenic event	Date (mya)	Range (mya)
Split of Empetrichythinae and Goodeinae	18.02	14.3–22.17
Split *Empetrichythy*s from *Crenichthys*	6.88	4.35–9.48
Split of Ilyodontini	14.05	11.62–16.79
Split of Girardinichthyini and Goodiini	10.84	8.97–12.68
Split of *Characodon*	13.12	10.82–15.53
Split of *Ilyodon* and *Xenotaenia* from *Allodontichythys*	8.44	5.85–11.26
Split of *Goodea* and *Ataeniobius*	9.30	6.50–11. 99
Split of *Allotoca*	9.94	8.29–11.74
Split of Chapalichthyini	11.43	9.59–13.32

### Goodeidae variation in body shape

The tests for overall body shape differences between the two subfamilies revealed significant differences compared to a Brownian motion model of evolution (Table 2). Specifically, the estimated rate of body shape evolution for the Goodeinae was almost twice as high as that for the Empetrichthyinae (σ ^2^_E_ = 9.84 × 10^− 4^ vs. σ ^2^_G_ = 1.90 × 10^− 3^).

**Table 2:** Evolutionary shape rate results and Modular Evolutionary Rate Results, with associated *p* values for significance testing. Test statistics include σ^2^_R_ for the evolutionary shape rate ratio between the two subfamilies, Rmult is the module shape rate ratio between different modules, and the subscripts "E" and "G" represent Empetrichthyinae and Goodeinae, respectively

Comparison	σ^2^_R_	P	σ ^2^_E_	σ ^2^_G_
Body Shape				
LM 1–18	1.94	0.041	9.839 × 10^−4^	1.900 × 10^−3^
Head Shape				
LM 1–12	1.564	0.15	2.368 × 10^−3^	3.730 × 10^−3^
Tail Shape				
LM = 9–14	2.51	0.021	5.567 × 10^−3^	1.397 × 10^−2^
Comparison	R_mult_	P	σ ^2^_B_	σ ^2^_A_
Modular Traits				
A = LM 1–8, 15–18	2.556	0.0007	0.002737	0.001071
B = LM 9–14			2.74 × 10^−3^	1.07 × 10^− 3^

### Modularity within the Goodeidae

When the morphometric data were further divided into functional/locomotor traits (head and trunk landmarks), only the trunk region showed a significant difference between the subfamilies, (σ^2^_R_^Head^ **=** 1.564 *p* = 0.150, σ^2^_R_^Trunk^ **=** 2.510 *p* = 0.021), indicating that these phenotype modules are evolving at different evolutionary rates within the Goodeidae. The head region did not show any significant differences in evolutionary rate between the two subfamilies, suggesting that the Empetrichthyinae and Goodeinae have not diversified along trophic axes.

The results from a comparison of only evolutionary trunk shape rate change, corresponding to the habitat diversification hypothesis, indicate that the taxa with Goodeinae displayed over two and a half times more trunk shape evolution than the Empetrichthyinae (σ ^2^_E_ = 5.56 × 10^− 3^ σ ^2^_G_ = 1.39 × 10^− 2^).

### Body shape diversity within Goodeidae

**Fig. 3:**
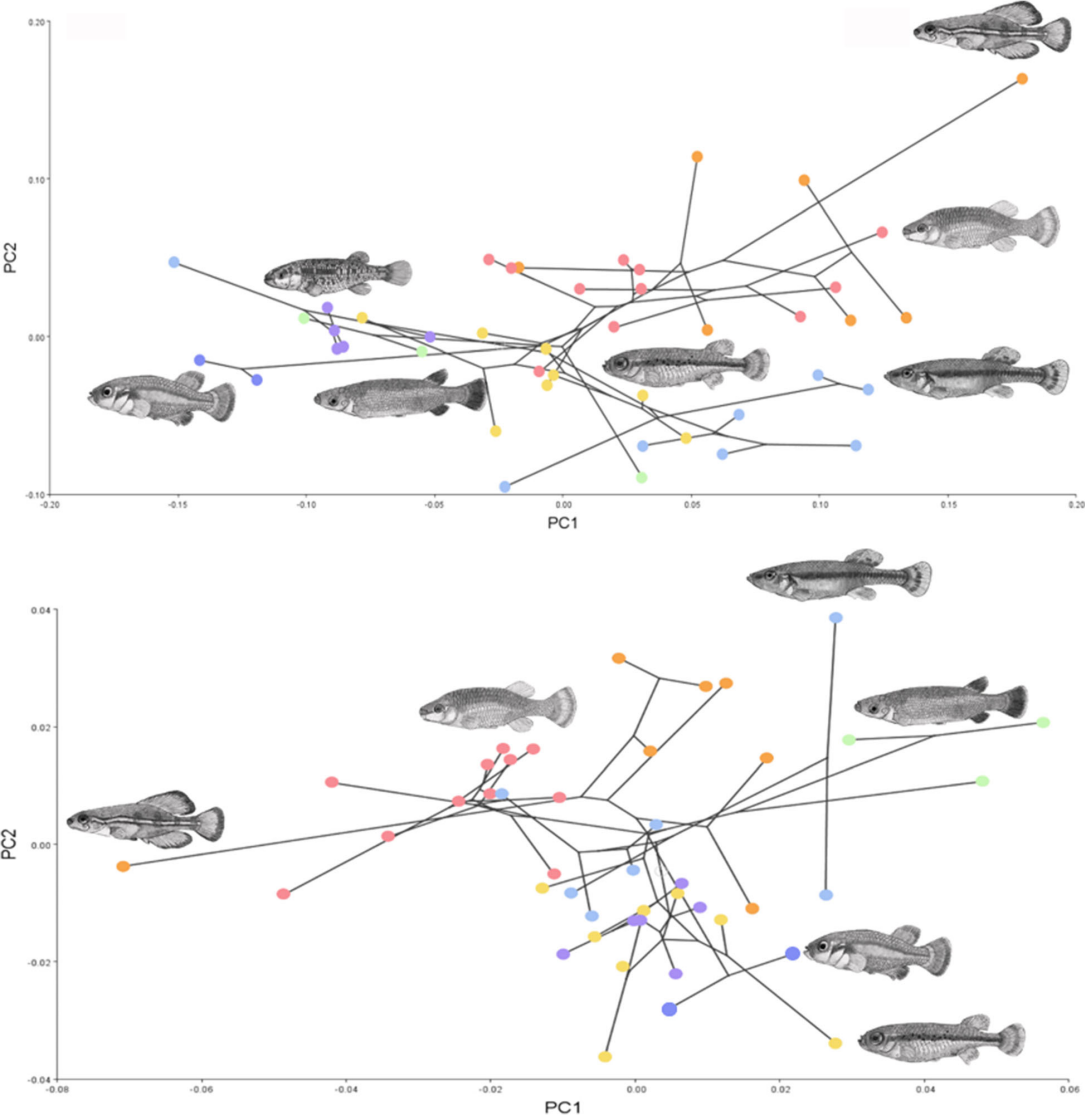
Phylomorphospace plots for all goodeid species from the time-calibrated phylogeny. Colors correspond to the seven clades recovered in Fig. 2. In particular, purple colors represent the Empetrichthyinae, whereas other colors represent the other groups of the Goodeinae. Phylomorphospace plots based on; **a**) tail shape, and **b**) overall body shape

The goodeids display a wide diversity of overall body shapes. When the extant taxa were plotted in phylomorphospace, the subfamily Goodeinae reaches into novel areas of morphospace for both overall (Fig. 3a) and trunk body shape plots (Fig. 3b). However, there is overlap among spring inhabiting species from both the Empetrichthyinae (*Empetrichthys latos, Crenichthys baileyi,* and *C. nevadae*) and Goodeinae (*Allodontichthys hubbsi, A. polylepis, and A. tamazulae*), which seem to independently have converged on a similar body shape in allopatry. Both PCs (1 and 2) showed the most shape change in the caudal peduncle and in the placement of dorsal and anal fin. PC1 (variance_Overall_ = 33%, variance_Trunk_ = 47%) was associated with a widening or compressing of the trunk area, with negative values associated with a widening of the dorsal lateral axis, and positive values are associated with a compression of the area. Positive PC2 (variance_Overall_ = 18%, variance_Trunk_ = 21%) values are associated with an elongation of the caudal peduncle and negative PC2 values are associated with a shortening of the caudal peduncle.

## Discussion

Disparity of species diversity within and between clades has been heavily studied [1, 2, 10, 46]. As stated earlier, most studies of diversification have focused on trophic specializations [47–49] or habitat-based influences [11, 50–53] as the main drivers of diversification. Disentangling these phenomena (trophic- or habitat-based) has been challenging, but we now have the analytical tools to differentiate between these influences [54, 55].

Ecological opportunity or the evolution of a key innovation are two of the more common triggers of a diversification event in adaptive radiation scenarios [23, 55]. Historically, much of what is known about adaptive radiations was based on a few select and well-studied examples in isolated habitats such as oceanic islands, post-glacial lakes, and other unique and isolated environments [23, 56, 57]. However, the number of adaptive radiation examples has expanded dramatically in recent years, such that adaptive radiations no longer appear to be uncommon phenomena [58, 59].

Until recently, the correlation of morphological diversity and increased disparity, although a hallmark of adaptive radiations [27, 60, 61], has been difficult to test. Net differences in species diversification rates [62–64], examination of univariate phenotypic traits [25, 65, 66], and quantification of trophic based morphological differences [67–69] between clades have been utilized most often to test for influences on species diversification. These approaches ignore whole body shape or morphological associations as a whole (modules) and cannot provide the clearest picture of diversification. Linking innovations to increased speciation rates represent one of the strongest approaches for studying diversifications and adaptive radiations.

The disparity in species richness between the subfamilies Goodeinae and Empetrichthyinae offered a rare chance to test for contributors to lineage diversification. By utilizing geometric morphometrics, phylogenetic hypotheses, and phylogenetic comparative methods [51, 70], this study addressed two principle questions. First, does higher species diversity within the Goodeinae correlate with higher rates of morphological shape evolution in comparison to the Empetrichthyinae. The results from this study show a higher rate of overall body shape evolution in the Goodeinae that is almost double in magnitude than in the Empetrichthyinae. These results support the hypothesis that Mexican goodeids radiated in the Mesa Central, in a short time frame, as evidenced by their high rate of shape evolution, relative to the Empetrichthyinae. Additionally, the phylomorphospace plots show that many members of the Goodeinae reach into novel areas of morphological trait space and are often not clustered phylogenetically, whereas, the Empetrichthyinae did not expand into novel trait space and did not diversify to the same extent as the Goodeinae. Alternatively, the evolution of viviparity also could have contributed to the adaptation of the Goodeinae in these novel environments, followed by morphological diversification. The viviparous life-history of the group could have freed them being linked to particular substrate or structure for flow regime for spawning and egg attachment, thereby allowing them to diversify in this region.

The Mesa Central, the center of Goodeinae diversification, is an otherwise faunally depauperate, isolated highland plateau that has experienced hydrological compartmentalization events due to volcanism and orogeny during the Pliocene and Pleistocene [71]. One other group of fishes has diversified in the Mesa Central (*Chirostoma*: Atherinopsidae) via an adaptive radiation [71–73]. The Mesa Central, and the availability of an unoccupied array of heterogeneous habitats resulted in species of Goodeinae being were able to inhabit varying hydrological regimes from slow to fast flows throughout the Rio Lerma basin [34]. Although this has been speculated as contributing to Goodeinae diversification [35, 74], this idea previously has not been tested, as other studies have focused on the patterns of diversification within the Goodeinae [37] or for select groups within the subfamily [75].

The second objective of this study specifically addressed whether there are differences in trophic/locomotor modules between the two subfamilies, thereby testing whether the differences in disparity between goodeid lineages is the result of trophic or habitat specialization. Phenotypic trait modules can give insights into the developmental, functional, and locomotory influences of morphological disparity. Contrasting what is generally expected, the modularity rate tests within the Goodeidae (Goodeinae+ Empetrichthyinae) recovered a much higher rate of trunk region evolution than the head region. In general, goodeid species in both subfamilies are generalists in terms of feeding niches, and the majority of the species seem to be opportunistic omnivores [34]. This could explain why head shape showed no significant difference in evolutionary shape rates between the two clades of goodeid fishes. In general, diversification along trophic axes is a common phenomenon, especially for fishes. For example, east African Rift Lake cichlids have long served as a model in our understanding of diversification and adaptive radiation [76]. The large range in both species and phenotypic diversity within cichlids has been hypothesized to be a result of utilization of different dietary components [77–80]. In several cichlid groups, morphological structures have been linked to trophic diversity, such as the pharyngeal jaw and certain muscular elements of the jaw [81, 82], and are believed to allow for increased diversification because of trophic partitioning [83]. Studies have investigated diversification shifts within the group [77, 84, 85], or have examined the correlation between morphology and ecomorphological variables [68] to explain species disparity. Similarly, pupfish radiations also have been linked to differences in head morphology by assigning functional variables a priori and comparing rates of diversification [26].

When evolutionary shape rates of the trunk region were compared between the subfamilies, the Goodeinae showed a relative rate increase of over two-fold, in comparison to the same trunk region of the Empetrichthyinae using the approach outlined in [58]. Goodeinae taxa have diversified into various aquatic systems (canals, streams, rivers, lakes, ditches, and outflows) [34], whereas the Empetrichthyinae are confined to homogeneous habitats including small springs and pools [86]. The higher rate of trunk region evolution in Goodeinae may in fact show a constraint release of the phenotypic liability of the trunk region due to the Goodeinae experiencing different hydrodynamic pressure than the Empetrichthyinae.

Similar to this study, other studies have recovered a strong link between hydrodynamic flow and body shape [87, 88, 89–92]. Therefore, for fish species occupying a wide range of different hydrodynamic regimes (i.e. lakes, rivers, streams, springs), such as the Goodeinae, the variation in overall body shape, and more specifically, the trunk region of the body is expected to increase relative to close relatives occupying more homogeneous habitats. The modularity data supports the idea that such variation may be the result of the functional constraints of life in new hydrologic regimes (ecological release, sensu 71), as a result of ecological opportunity on the Mesa Central, thereby allowing for an increase in ecological niche space. All species within the Empetrichthyinae occupy spring and pool habitats and show little variation in trunk morphology and a low rate of trunk shape evolution.

## Conclusions

The results from this study support the hypothesis that the Goodeinae radiated via an ecological opportunity scenario (sensu 74), along habitat axes. The Goodeinae occupy the faunally depauperate, island-like Mesa Central. This is relevant because others have suggested that adaptive radiations primarily occur on islands or island–like habitats [75], although there are examples of radiations occurring under non-island like conditions [93]. The higher rate of trunk shape evolution for the Goodeinae supports the hypothesis that the Goodeinae ecological release following colonization of a diverse array of novel, unoccupied habitats during the Plio-Pleistocene facilitated directional selection on few key survival traits. It is likely that the diverse array of aquatic habitats and niches were either already occupied by competitors or not available for the Empetrichthyinae during the formation of the Great Basin, therefore both the species diversification and morphological diversification rates of the Empetrichthyinae were lower in comparison to the Goodeinae. Finally, the dated phylogeny suggests that there was rapid speciation and morphological diversification within the Goodeinae, lending additional support to the ecological opportunity hypothesis. Unlike most other studies of adaptive radiation, however, this study was able to disentangle the drivers of evolution and quantitatively eliminate trophic specialization as a driving force within the Goodeidae.

## Methods

### Phylogenetic analysis

Most mitochondrial DNA sequences of cytochrome *b* (cyt*b*) were obtained from Genbank (~ 1140 bp, http://www.ncbi.nlm.nih.gov/Genbank/) for the Goodeinae [33] and outgroup taxa (Additional file 1: Table S1). Additional sequences were generated in this study and tissue samples for these species were obtained from colleagues, natural history collections, or natural resource agencies. Sequence data were pruned to include one representative individual for each species. For the subfamily Empetrichthyinae, tissue samples were obtained, and DNA was extracted from fin clips using the DNeasy Blood and Tissue kit (Qiagen) following the manufacturer’s instructions. The cyt*b* gene was amplified via polymerase chain reaction (PCR) with the primers L14724 and H1595 from [94].

Each 25 μl cyt*b* PCR reaction consisted of the following: 0.75 μl MgCl_2_, 2.5 μl 10× buffer, 0.5 μl NTP, 0.5 μl of each 10 μM primer, 0.25 μl *Taq*, 1 μl of DNA template, and 19–20 μl of water. The thermal cycling parameters for cyt*b* were as follows: 94 °C for 2 min, 27 cycles of 94 °C for 45 s, 54 °C for 30s, 72 °C for 1 min, and a final extension of 74 °C for 10 min. DNA sequencing was performed by the Beckman Coulter Genomics Facility (Danvers, MA). Alignment and editing of sequences was performed using Geneious 9.1.4 [95] Sequence data were submitted to GenBank (Additional file 1: Table S1).

Beast v1.8 [96] was used to generate a time-calibrated phylogeny, using the General Time Reversible (GTR) model, a relaxed lognormal clock, and a tree prior of Yule Process [97]. Divergence time estimations were conducted using eight fossil calibrations, lognormal priors, and minimum time estimates based on the oldest known fossils of select ingroup and outgroup taxa (Table 3). Two separate runs consisting of 40 million generations each were run sampling every 1000 generations. Tracer V1.5 [98] was used to determine effective sampling size (ESS) of all parameters and to determine that the analysis reached stationary. All ESS parameters were ˃200, indicating that the parameters robustly sampled the posterior distribution. A maximum clade credibility tree using a 20% burn-in was created with the program Tree Annotator V1.8.3 [96] and was used as the backbone for phenotypic diversification analyses.

**Table 3:** Fossil calibrations and parameters used in BEAST to construct a dated phylogeny

Fossil	Offset	Mean	StDev	Citation
A) †*Polymixia sp.*	95	2	1	McMahan et al. 2013 [85]
B) †*Gymnogeophagus eocenicus*	46	1.95	1	McMahan et al. 2013 [85]
C) †*Plesioheros* and *†Tremembichthys*	40	1.88	1	McMahan et al. 2013 [85]
D) Cyprinidontiformes	55	6.4	1	Marchio and Piller 2013 [111]
E) Poeciliidae and Anablepidae	40	2.05	1	Santini et al. 2009 [112]
F) Fundulidae	13.2	2.45	1	Bickley 1970 [113]; Cvancara et al. 1971 [114]
G) Empetrichthyinae	3.6	2.56	1	Jordan 1923; Uyeno and Miller 1962 [115]
H) Goodeidae	9	2.5	1	Alvarez and Arriola-Longoria 1972 [116]

The symbol "†" refers to fossil genera

### Geometric Morphometrics

**Fig. 4:**
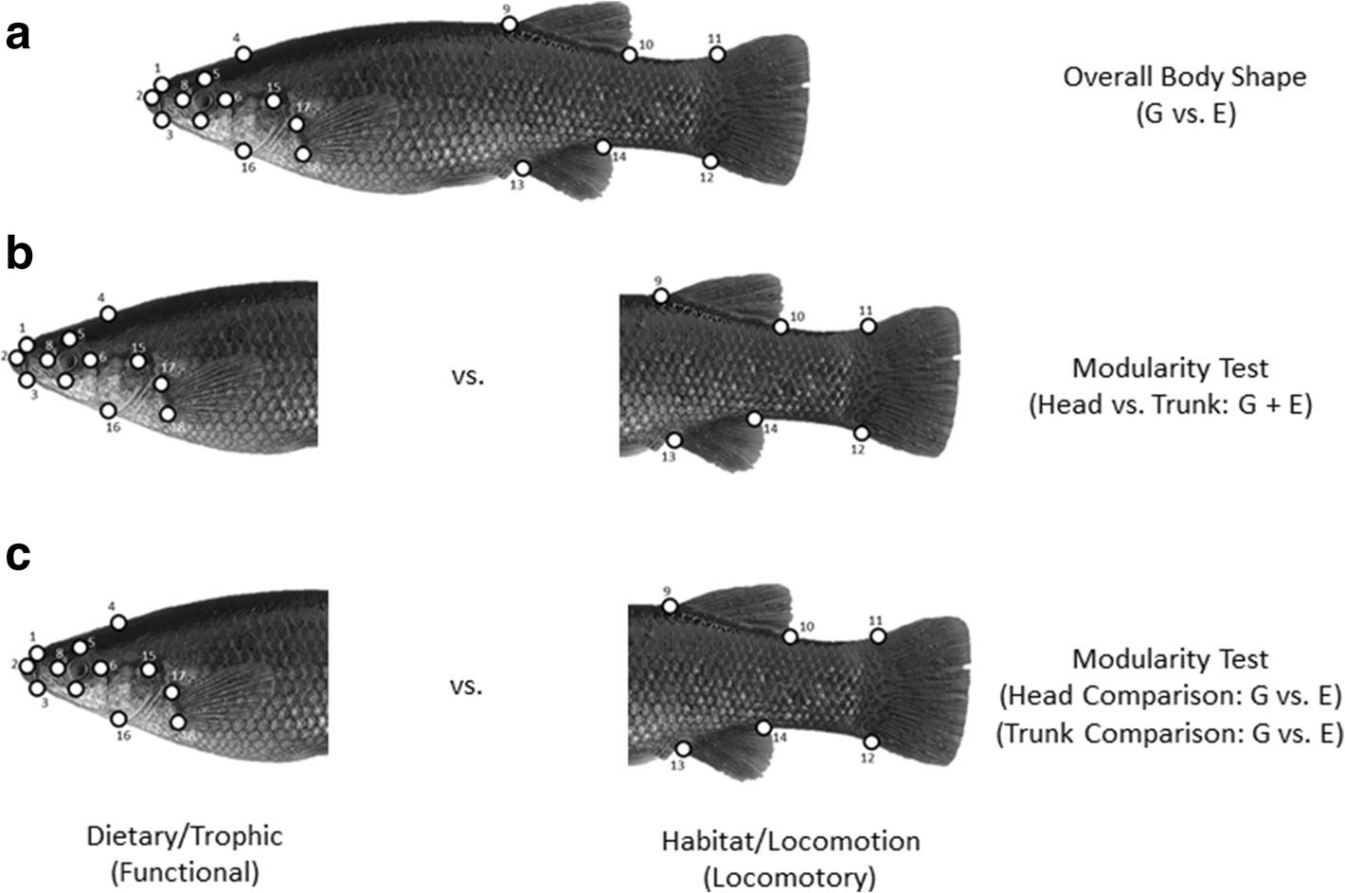
**a** Distribution of landmarks used for geometric morphometric analyses and evolutionary shape rate tests, **b** modularity rate test comparing head and trunk morphologies of the Goodeinae [G] versus the Empetrichythinae [E], and **c**) modularity rate test by body region testing the trophic diversification hypothesis versus habitat diversification hypothesis by subfamily

Images of museum specimens (*N* = 1669) of all species (40 species and 4 subspecies) were taken from the left lateral side using a Nikon D5000 SLR camera and a 50 mm macro lens. Museum abbreviations follow [99]. Bent, warped, or juvenile specimens were not utilized to limit ontogenetic effects and non-biological variation. Eighteen homologous, two dimensional landmarks were used to summarize body shape [100] using the program TPSdig version 2.12 [101]. The landmarks (Fig. 4a) included; 1) anterior tip of the snout, 2) opening of mouth, 3) posterior edge of jaw, 4) posterior edge of the neurocranium, 5) upper edge of eye, 6) posterior edge of eye, 7) ventral edge of eye, 8) anterior edge of eye, 9) anterior edge of the dorsal fin, 10) posterior edge of the dorsal fin, 11) dorsal edge of the caudal fin, 12) ventral edge of the caudal fin, 13) anterior insertion of the anal fin, 14) posterior insertion of anal fin, 15) intersection of gill opening and ventral margin of body, 16) posterior most edge of neurocranium, 17) upper insertion of the pectoral fin, and 18) lower insertion of the pectoral fin.

### Rate of body shape evolution analyses

We used the multivariate rates methodology developed by [102] to examine rates of body shape evolution using all of the landmarks. This approach utilizes geometric morphometric data and a phylogeny to test for differences in evolutionary shape rates (σ^2^). This method uses multivariate distances and a Brownian motion model of evolution that assumes that variance increases with time. The distance-based approach has also been shown to be statistically robust when increasing trait dimensions, which is unavoidable in multivariate data [103].

A Procrustes superimposition analysis was carried out in the program MorphoJ [104], which corrects for scaling, rotation, translation, and size biases. All other analyses were performed in R [105] using the packages ‘geomorph’, ‘phytools’, and ‘geiger’. Mean shape data from the Procrustes data were calculated for each species for the shape analyses. The time-calibrated phylogeny was pruned to include only the taxa for which morphological data were available using the ‘drop.tip’ function in the package ‘phytools’. The multivariate technique for testing evolutionary rates (σ^2^) using the shape data collected and the time-calibrated phylogeny was implemented [102]. The evolutionary shape rate for each subfamily was recovered (σ^2^_G_, σ^2^_E_), along with a ratio for Goodeidae and Empetrichthyinae (σ^2^_R_), which represents relative differences in evolutionary shape rate between the two subfamilies. Phylogenetic simulation of 9999 iterations was used to test the null hypothesis that one single rate is found between the two subfamilies.

We used the procedures in [106] and [102] to test for multivariate differences between sets of phenotypic traits (modules). Modules are sets of traits that show highly mingled connections to each other, but are loosely connected to other sets of traits [107, 108]. Modules have long been thought to vary in evolutionary rates; for example, the steady swimming hypothesis predicts caudal peduncle ratio differences among individuals in fast vs. slow moving aquatic systems [87, 88].

We conducted a modularity test to specifically test for differences in trophic/locomotor modules (head vs. trunk regions), corresponding to the trophic and habitat diversification hypotheses (Fig. 4b). The head region consisted of landmarks 1–8, 15–18, whereas the trunk region included landmarks 9–14 (Fig. 4c). This test was accomplished using a two-step approach incorporating the Procrustes aligned shape data considered by module, along with a time-calibrated phylogeny under a Brownian model of evolution. First, we compared evolutionary shape rates between the two phenotypic modules (head vs. trunk) (Fig. 4b) to determine whether the modules are evolving at different rates, and therefore represent independent anatomical units. Second, we then compared evolutionary shape rates within each module by subfamily to determine if there were rate differences at the subfamily level within each module. The procedures follow [106] and were carried out in the package ‘geomorph’. Landmarks were divided by morphological regions, and then tested using relative rate ratios.

Finally, we used the phylomorphospace approach [109, 110] to characterize the evolutionary patterns of body shape diversity within the Goodeidae (overall, and within phenotypic modules). This multivariate phenotypic method projects the phylogeny and the first two principal components of a principal components analysis of the aligned morphometric data to depict the evolutionary history of morphospace occupancy for the goodeid fishes.

## Additional file

Additional file 1: **Table S1.** Genbank accession numbers and locality information used to generated the time calibrated phylogeny. (XLSX 13 kb)
